# Two Pregnancies with a Different Outcome in a Patient with Alport Syndrome

**DOI:** 10.3889/oamjms.2016.073

**Published:** 2016-07-02

**Authors:** Biljana Gerasimovska Kitanovska, Vesna Gerasimovska, Vesna Livrinova

**Affiliations:** 1 *University Clinic of Nephrology, Ss Cyril and Methodius University of Skopje, Skopje, Republic of Macedonia*; 2 *University Clinic of Gynecology and Obstetrics, Ss Cyril and Methodius University of Skopje, Skopje, Republic of Macedonia*

**Keywords:** Alport syndrome, chronic kidney disease, pregnancy

## Abstract

**BACKGROUND:**

Alport syndrome is a genetic disease that progresses to chronic kidney failure, with X-linked, autosomal dominant or autosomal recessive type of inheritance. Women are generally carriers of the mutation and have a milder form of the disease. During pregnancy, they have an increased risk of impaired kidney function and preeclampsia.

**CASE PRESENTATION:**

A 27-year old woman, gravida 1, para 0, in her 23rd gestational week came to the outpatient unit of the University Clinic of Nephrology for the first time because of slowly progressing proteinuria and Alport syndrome. She was admitted to the gynaecological ward in her 29th gw for proteinuria which increased from 3.8 g/day up to 20 g/day and the serum creatinine increased to 120- 150 micromol/l. She was delivered in the 30th gestational week due to obstetrical indications with a cesarian section and delivered a baby with a birth weight of 880 g. After delivery, proteinuria decreased to 2 g/d within 2 months and an angiotensin-converting enzyme inhibitor (ACEI) was started. Her second pregnancy, after 2 years, had an uneventful course and she delivered a healthy baby weighing 3000 g in the 39th week. Six months after the second delivery, her renal function remained normal and her proteinuria was 2 g/d.

**CONCLUSIONS:**

Pre-pregnancy counselling and frequent controls during pregnancy are necessary for women with Alport syndrome, as well as regular monitoring after delivery. Recent reports are more in favour of good pregnancy and nephrological outcomes in women with Alport syndrome when renal disease is not advanced.

## Introduction

Alport syndrome is a genetic disease that progresses to chronic kidney failure and is characterised by persistent hematuria, proteinuria and progressive renal disease. It may be associated with hearing loss and ocular abnormalities, but not all clinical characteristics are found in all.patients with Alport syndrome [1].

It is associated with mutations in type IV collagen gene and may have an X-linked (mutation on *COL4A5*), autosomal dominant (mutation on *COL4A3* or *COL4A4*) or autosomal recessive type of inheritance (mutation on *COL4A3* or *COL4A4*) [2].

Women are generally carriers of the mutation and have a milder form of the disease, with slow progression of the renal disease [3]. During pregnancy, they have an increased risk of impaired kidney function and preeclampsia [4]. There are only several case reports found in the literature on Alport syndrome and pregnancy.

## Case Presentation

A 27-year old woman, gravida 1, para 0, in her 23^rd^ gestational week consulted at the outpatient unit of the University Clinic of Nephrology in Skopje, Macedonia, for the first time, because of slowly progressive proteinuria.

Her father was diagnosed with Alport syndrome with renal biopsy, had chronic kidney failure, was on a hemodialysis and died at the age of 57. Her uncle also had Alport syndrome confirmed by biopsy and was on hemodialysis. The patient and her two sisters were diagnosed with Alport and renal biopsy was not done because of their positive family history. They were followed since their childhood for erythrocyturia and different levels of proteinuria, but with normal serum creatinine and no hypertension. Her older sister, who has only erythrocyturia and no proteinuria, had a successful pregnancy with a term birth of a child with slightly lower birthweight. Her younger sister has erythrocyturia and proteinuria above 4 g/d. Unfortunately, genetic analysis is not available in the country and was not done in our patient.

Proteinuria was first registered in our patient 5 years before her first pregnancy, at her age of 22 and was in the range of 0.4 – 0.8 g/d.

### First pregnancy

When the patient consulted in the Department of Nephrology in the 23rd gw, she had higher serum creatinine for pregnancy, 89 micromol/l, and in the urine, there were 20-25 erythrocytes in the sediment - 17% of which were dysmorphic. Her renal ultrasound revealed an ectopically placed right kidney and hypertrophy of her left kidney. She was started on Aspirin 100 mg from the onset of the pregnancy. The 24-hour proteinuria was 0.6 g/dU and progressed over time, and hypoalbuminemia worsened. Her D-dimers in the 23rd gw were slightly increased and low-molecular-weight heparin was started at a dose of 40 g.

In 26 the gw, intrauterine growth retardation (IUGR) was registered and the fetus was bio-metrically equal to 23 rd gestational week. There was already an increased resistance index (RI) at the umbilical artery up to 0.71 and a verified notch in the left uterine artery. In the 29th gw, the fetus was bio-metrically equal to 25th gw, with a notch in the right umbilical artery. Blood pressure was within normal.

She was admitted to the gynaecological ward in her 29th gw for proteinuria which increased from 3.8 g/day up to 20 g/day and the serum creatinine increased to 120- 150 micromoles/l. Her albumin was 29-22 g/l and uric acid was 332 micromoles/l. Blood pressures increased up to 140/80 mm Hg and she was started on Methyldopa 500 mg and continued with 250 mg. She was started on a small dose of corticosteroids in order to stimulate lung maturation.

She was delivered in the 30th gestational week due to obstetrical indications with a cesarian section and delivered a baby with a birth weight of 880 g. The baby died within 7 days and autopsy showed that respiratory distress was the cause of death.

After delivery, proteinuria decreased to 2 g/d within 2 months and angiotensin-converting enzyme inhibitor (ACEI) was started. Because of a persistent cough, she was switched to an angiotensin-receptor blocker (ARB), Losartan 25 mg/d and her proteinuria decreased to 0.8 g/d. Her blood pressure remained normal and serum creatinine was 70-83 micromol/l at the follow-up two years after delivery.

### Second pregnancy

After two years of her first pregnancy, the patient became pregnant again, with a baseline proteinuria of 2 g/dU and serum creatinine 89 micromol/l. Her second pregnancy was uneventful, with regular monthly checkups, her blood pressure was in normal range and her proteinuria increased from 2 g/d up to 3.5 g/d by the 39^th^ gestational week. Her serum albumin was 29 g/l at the 39^th^ gestational week and creatinine rose to 81 g/l. She delivered a healthy baby by cesarian section in her 39^th^ gestational week. Six months after the second delivery, her renal function remained normal and her proteinuria was 2/d.

## Discussion

Chronic kidney disease (CKD) used to be a contraindication for pregnancy. It is no longer considered to be so, yet if the kidney disease is in an advanced stage, it is more difficult for a patient to conceive and have a successful pregnancy [4]. In the management of CKD in pregnancy, it is more important to manage clinical features than to know the aetiology [5]. Intrauterine growth retardation and preterm birth, as well as the risk for worsened kidney disease, occur twice more often in pregnant women with the renal disease. The risk from decreased renal function in pregnancy should be considered separately from comorbidities that may aggravate the condition [6]. A case-control study has shown that when proteinuria is below 1 g/d, serum creatinine is below 110 micrograms/d, without hypertension, pregnancy did not have an adverse effect on long-term kidney function [7].

Alport syndrome in women is generally a mild disease, but there is a wide variability of renal outcomes. It is suggested that in heterozygous females with Alport syndrome, inactivation of the X chromosome may be important for the severity of the disease. Tissue-specific distribution of an alternatively spliced *COL4A5* isoform and non-random X chromosome inactivation reflect phenotypic variation in heterozygous X-linked Alport syndrome [8].

In the first pregnancy, our patient had chronic kidney disease in stage 1 from the beginning of pregnancy (erythrocyturia, albuminuria). Her serum creatinine was within normal, proteinuria was below 1 g/d and her blood pressure was normal so that an uneventful pregnancy was expected. Yet, worsening proteinuria and hypoalbuminemia were associated with intrauterine growth retardation and biometrically smaller fetus, and despite corticosteroids for lung maturation, and low-molecular weight protein prophylaxis, the fetus had birthweight of 880 g and died due to respiratory distress. In the following period after delivery, the patient was followed at regular intervals and proteinuria decreased due to ACEI. In her next pregnancy, proteinuria started from 2 g and at the end of the pregnancy it increased to 3.5 g/dU, yet it was not the reason for a pre-term delivery nor intrauterine growth retardation. After delivery, the patient’s renal function did not deteriorate further.

There are only several case reports on the outcomes of pregnancy in women with Alport syndrome. Previous reports showed that pregnancies in women with Alport syndrome ended with preeclampsia and worsened renal function. Matsuo described a case with higher proteinuria (1-2 g/d) and higher serum creatinine from the start of the pregnancy, which evolved into preeclampsia and worsened kidney function [9]. He speculated that preeclampsia is more frequent in women with Alport syndrome because type 4 collagen can be found in placental and renal vessels and in the case of a mutation in both sites, destruction may occur and a common antigen may be involved [10]. Still, the report by Matsubara showed that umbilical cord of a newborn from a mother with Alport had negative immunofluorescence staining for the alpha 5 chain of type IV collagen. A good pregnancy outcome is reported by Matsoubara [11, 12] and Alessi [13]. Recent expert guidelines on Alport syndrome state that ACEI should be used between pregnancies because they are nephroprotective and that they would be beneficial for proteinuria, and other risk factors for renal failure progression in patients with Alport syndrome [14].

The risk for preeclampsia and worsened renal disease is increased in pregnant women with Alport syndrome [15]. Recent reports present cases with more favourable outcomes in both autosomal dominant and recessive cases [16, 17].

Considering the outcomes of pregnancies in women with Alport syndrome, pre-pregnancy counselling and frequent controls during pregnancy are necessary, as well as regular monitoring after delivery. Prepregnancy counselling should include information for the parents on the risk for the syndrome in the child and offered a prenatal diagnosis in the fetus. Controls during pregnancy should be once monthly, once a week immediately after delivery and then once a month in the first six months, their frequency in the next period depending on the renal affection. Recent reports are more in favour of good outcomes of pregnancies in women with Alport syndrome when renal disease is not advanced.
